# Terbinafine and Neutropenia

**DOI:** 10.4274/tjh.2014.0486

**Published:** 2015-05-08

**Authors:** İrfan Yavaşoğlu

**Affiliations:** 1 Adnan Menderes University Faculty of Medicine, Division of Hematology, Aydın, Turkey

**Keywords:** Terbinafine, Drug, Neutropenia

## TO THE EDITOR

The article entitled “Aplastic Anemia Associated with Oral Terbinafine: A Case Report and Review of the Literature”, written by Kantarcıoğlu et al. and published in a recent issue of your journal, was quite interesting [1]. Here we would like to emphasize some relevant points.

In the assessment of 425 cases by van der Klauw et al., the most common causes of drug-related agranulocytosis or neutropenia were, in order, dipyrone, mianserin, sulfasalazine, trimethoprim-sulfamethoxazole, penicillins, cimetidine, thiouracil groups, and phenylbutazone [2]. Terbinafine was not included in this list. Neutropenia associated with terbinafine is more common in women, which may be due to more fungal infections encountered and more drug usage for this purpose. To know the MCV and vitamin B12 levels in the presented patients would be useful.

In our case, a patient presented with neutropenia due to terbinafine. A 64-year-old man with a history of onychomycosis presented with neutropenia after starting terbinafine at 250 mg/day [3]. In conclusion, routine hematological monitoring is not indicated, but patients should be informed of this potentially life-threatening adverse reaction.

**Conflict of Interest Statement**

The author of this paper has no conflict of interest, including specific financial interests, relationships, and/or affiliations relevant to the subject matter or materials included in this manuscript.
