# Supplementary material for: Terbinafine and Neutropenia
Source: Turk J Haematol. 2015 May 8;32(2):189. doi: 10.4274/tjh.2014.0486 (PMC4706084; doi:10.4274/tjh.2014.0486)
Supplement: Related texts

# Reply: Authors' Response

**Authors:** Buent Kantarcıoglu

## REPLY

We are very pleased by the interesting comment and participation by Yavaşoğlu. As we already mentioned in the article oral terbinafine is a highly effective agent, which is generally well tolerated. Two large-scale postmarketing surveillance studies showed that the incidence of serious side effects was [1] [1,2]. We want to clarify that the main purpose of our report was to share the experience of handling such a difficult case with colleagues who are practicing medicine, since the definite causality of aplastic anemia with oral terbinafine cannot be established [3]. The well prepared case presented by Yavaşoğlu and Arslan is another example in this regard [4]. In addition MCV and serum vitamin B12 levels were missing in most of the cases reported in the literature, but they were both in normal ranges in our case. 

## References

[1] Hall M, Monka C, Krupp P, O’Sullivan D (1997). Safety of oral terbinafine: results of a postmarketing surveillance study in 25,884 patients.. Arch Dermatol.

[2] O’Sullivan DP, Needham CA, Bangs A, Atkin K, Kendall FD (1996). Postmarketing surveillance of oral terbinafine in the UK: report of a large cohort study. Br J Clin Pharmacol.

[3] Kantarcıoğlu B, Turkoz HK, Yılmaz G, Tanrıkulu FP, Atagunduz IK, Adiguzel C, Tuglular TF (2014). Aplastic anemia associated with oral terbinafine: a case report and review of the literature. Turk J Hematol.

[4] Yavaşoglu I, Arslanøj E (2008). ANeutropenia due to terbinafine. Osmangazi Tıp Dergisi.
